# Genome sequence of *Staphylococcus lugdunensis* N920143 allows identification of putative colonization and virulence factors

**DOI:** 10.1111/j.1574-6968.2011.02339.x

**Published:** 2011-07-04

**Authors:** Simon Heilbronner, Matthew TG Holden, Andries van Tonder, Joan A Geoghegan, Timothy J Foster, Julian Parkhill, Stephen D Bentley

**Affiliations:** 1 Microbiology Department, Trinity CollegeDublin, Ireland; 2 The Wellcome Trust Sanger Institute, Wellcome Trust Genome CampusHinxton, Cambridge, UK

**Keywords:** *Staphylococcus lugdunensis*, genome sequence, surface proteins, virulence factors

## Abstract

*Staphylococcus lugdunensis* is an opportunistic pathogen related to *Staphylococcus aureus* and *Staphylococcus epidermidis*. The genome sequence of *S. lugdunensis* strain N920143 has been compared with other staphylococci, and genes were identified that could promote survival of *S. lugdunensis* on human skin and pathogenesis of infections. *Staphylococcus lugdunensis* lacks virulence factors that characterize *S. aureus* and harbours a smaller number of genes encoding surface proteins. It is the only staphylococcal species other than *S. aureus* that possesses a locus encoding iron-regulated surface determinant (Isd) proteins involved in iron acquisition from haemoglobin.

## Introduction

*Staphylococcus lugdunensis* is a coagulase-negative *Staphylococcus* (CoNS). It is a commensal of the human skin that is found more frequently on the lower part of the body and the extremities, particularly in moist areas such as the perineum, the inguinal fold and under the large toenail (Bieber & Kahlmeter, 2010). Although it is regarded as an integral part of the normal human biota*, S. lugdunensis* is an opportunistic pathogen, causing serious skin and soft tissue infections, and is particularly associated with serious cases of infective endocarditis (IE), more akin to *Staphylococcus aureus*. Between 1% and 5% of IE cases are caused by *S. lugdunensis*. Thus, it is more virulent than expected for CoNS (Frank *et al.*, 2008).

It is conceivable that the incidence of infections caused by *S. lugdunensis* is under-reported. In the clinical microbiology diagnostic laboratory, it could easily be mistaken for *S. aureus* because of its colony morphology, haemolytic activity and ability to agglutinate latex particles coated with fibrinogen (Zbinden *et al.*, 1997).

The complete genome sequence of the *S. lugdunensis* strain HKU09-01 has been published (Tse *et al.*, 2010). However, the annotation is incomplete and an in-depth analysis of potential colonization and virulence factors has not been carried out. This paper describes conclusions that were drawn from analysing the genome sequence of *S. lugdunensis* N920143 and comparing it with that of HKU09-01.

## Materials and methods

### Bacterial strain

*Staphylococcus lugdunensis* N920143 was isolated from a breast abscess in 1992. It was kindly supplied by Dr F. Vandenesch, Université Lyon, Lyon, France.

### Genome sequencing

*Staphylococcus lugdunensis* N920143 genomic DNA was isolated using the PurElute Bacterial Genomic Kit (Edge Biosystems) with an additional incubation step with lysostaphin (25 μg mL^−1^) at 37 °C for 10 min. Genomic DNA was ethanol precipitated and dissolved in TE buffer for sequencing.

The genome of the *S. lugdunensis* strain N920143 was sequenced using both reversible terminator sequencing [on Illumina Genome Analysers (GAII)] and pyrosequencing (on 454 instruments; subsidiary of Roche Diagnostics Corporation, Branford, CT). A total of 40.1 Mb of Illumina sequence was produced from a 200-bp standard paired-end library run in one lane of a flow cell with 54-bp reads representing approximately 850-fold coverage. The 454 sequencing produced a 0.23-Mb sequence with an average length of 250 bp. The assembly of the Illumina reads using velvet 0.7.62 gave 142 contigs of >1 kb with a contig N50 of 22 kb.

A combined assembly of the 454 reads using newbler 2.1 and the Illumina consensus sequences from the velvet assembly produced 69 contigs >500 bp with an N50 of 72 kb. The length of the combined assembly was 2 588 004 bp in nine scaffolds. image (Tsai *et al.*, 2010) and icorn (Otto *et al.*, 2010), along with a further 1070 high-quality reads, were used to close gaps and to improve the quality of the sequence to the standard of improved high-quality draft (Chain *et al.*, 2009).

The sequence and annotation of the *S. lugdunensis* strain N920143 genome has been deposited in the EMBL database with the accession number FR870271. The sequence was finished and annotated as described previously using artemis software (Holden *et al.*, 2009). Comparison of the genome sequences was facilitated using the artemis comparison tool (act) (Carver *et al.*, 2005). Orthologous proteins were identified as reciprocal best matches using fasta (Pearson & Lipman, 1988) with subsequent manual curation.

## Results

### Comparative genomics

The genome of *S. lugdunensis* N920143 comprises an approximately 2.6-Mbp chromosome. The sequence consist of six contigs and accordingly six gaps of approximately 1.5 –4 kb, occurring in repetitive DNA stretches like rRNA and the repeat region of a surface-anchored protein. The contigs were aligned according to their order in HKU09-01. Gap closing was not completed because no important features are encoded within the regions in HKU09-01. The genome contains a single prophage named φSL1 and 14 insertion sequences. It does not carry any integrated or replicating plasmids. Our analysis has identified the genomic differences that distinguish *S. lugdunensis* from *S. aureus* and other CoNS, and as a corollary of this, has addressed how the genome may influence its biology and ability to cause disease.

**Fig. 1:**
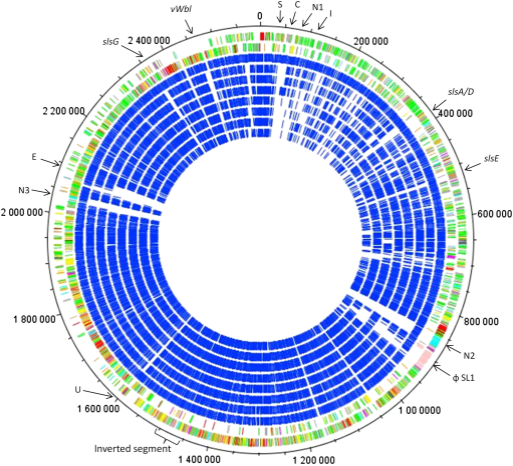
Schematic circular diagram of the *Staphylococcus lugdunensis* N920143 chromosome. Key for the circular diagram (outer to inner): annotated CDSs coloured according to predicted function are shown on a pair of concentric circles, representing both coding strands; blue circles show *S. lugdunensis* N920143 reciprocal fasta best matches shared with *S. lugdunensis* HKU09-01; MRSA252; MSSA476; *Staphylococcus epidermidis; Staphylococcus haemolyticus; Staphylococcus saprophyticus; Staphylococcus carnosus; Macrococcus caseolyticus*. Colour coding for *S. lugdunensis* CDS functions: neon green, pathogenicity/adaptation; dark gray, energy metabolism; red, information transfer; dark green, surface associated; sky blue, degradation of large molecules; dark pink, degradation of small molecules; yellow, central/intermediary metabolism; pale green, unknown; pale blue, regulators; orange, conserved hypothetical; brown, pseudogenes; pink, phage and IS elements; gray, miscellaneous. Arrows indicate regions of interest: C, CRISPR region; E, ESAT 6 toxin and secretion; I, Isd operon; N1, nonribosomal peptide synthetase 1; N2, nonribosomal peptide synthetase 2; N3, nonribosomal peptide synthetase 3; φSL1, prophage; S, streptolysin S-like toxin; U, gene cluster for sugar uptake and degradation.

Phylogenetic analysis of 16S rRNA gene (Takahashi *et al.*, 1999) and *dnaJ* (Shah *et al.*, 2007) gene sequences places *S. lugdunensis* in a clade that includes *Staphylococcus epidermidis, S. aureus* and *Staphylococcus haemolyticus*. Comparative genomic analysis revealed that a large proportion of the *S. lugdunensis* N920143 genome is shared with these related pathogenic staphylococci. Of the 2447 coding sequences (CDSs) in the N920143 genome, 77.8% have reciprocal fasta matches to *S. aureus* MRSA252, 74.7% to *S. epidermidis* RP62a and 78.3% to *S. haemolyticus*. In comparison with the more distantly related staphylococci, 71.4% of all CDSs have matches to *Staphylococcus saprophyticus*, 71.3% to *Staphylococcus carnosus* and 54.4% to *Macrococcus caseolyticus* (Fig. 1).

Compared with all other staphylococci, there is an inversion of 60 375 bp located between 1 457 500 and 1 518 000 in a conserved region of the genome. Analysis of the boundaries of the inversion in N920143 has failed to identify flanking repeat sequences that could account for the inversion via recombination.

A pairwise comparison between N920143 and HKU09-01 identified that 95.4% of the chromosome is conserved (including the inversion), and that there are 125 unique CDSs in N920143. The major differences between *S. lugdunensis* N920143 and HKU09-01 are (1) the presence of two identical putative transposons in HKU09-01 encoding a β-lactamase (*bla*) and β-lactamase regulatory proteins, (2) a putative genomic island in HKU09-01 encoding resistance to cadmium, but lacking identifiable virulence factors, (3) the gene encoding one of the three SLUSH peptides (C) is missing in HKU09-01 and (4) the duplication in HKU09-01 of a 32-kb region comprising the locus encoding the iron-regulated surface-determinant locus (Isd). The duplication occurred by unequal recombination between SLGD_00058 (annotated as KdpA, potassium-transporting ATPase A chain) and SLGD_00116 (annotated as Na^+^ driven multidrug efflux pump). These ORFs flank the ∼16-kp *isd* locus and are separated by 32 kb in N920143. Recombination occurred between 19-bp sequences within the ORFs that are identical apart from one mismatch to create a hybrid gene SLGD_00087 in HKU09-01 (annotated asKdpA potassium-transporting ATPase A chain).

### Putative virulence and colonization factors

We have identified several loci that might be of relevance to skin survival and virulence (Table 1). All staphylococci carry genes that encode a single iron-regulated ferric siderophore uptake system (*sst*) (Morrissey *et al.*, 2000), which is duplicated in both *S. lugdunensis* strains, an accessory gene regulator (Agr) system (Otto, 2001) and systems for neutralizing the negatively charged cell envelope by adding d-alanine to teichoic acid (Dlt) (Peschel *et al.*, 1999) and l-lysine to phosphatidyl glycerol (MrpF) (Peschel *et al.*, 2001). *Macrococcus caseolyticus* and all sequenced staphylococci except *S. haemolyticus* and *S. carnosus* have the capacity to express poly-*N*-acetyl glutamine encoded by the *ica* operon (Goetz, 2002). Only the bovine strain of *S. aureus* RF122 along with *S. lugdunensis* has a locus encoding a putative streptolysin S-like toxin (Lee *et al.*, 2008). *Staphylococcus lugdunensis* also carries a locus of four genes encoding lantibiotic resistance proteins similar to GdmG/E/F/H of *Staphylococcus gallinarum* (Siezen *et al.*, 1996). Interestingly, no lantibiotic biosynthesis proteins are encoded within the locus. Furthermore, *S. lugdunensis* encodes a polysaccharide (O'Riordan & Lee, 2004) and a polyglutamic acid capsule (Kocianova *et al.*, 2005), three nonribosomal peptide synthesis systems [one of the three synthetases is conserved in *S. aureus* and *S. epidermidis* (Wyatt *et al.*, 2010)] and an ESAT-6 toxin secretion system (*ess*) with homology to ESAT-6 proteins of *Mycobacterium tuberculosis* (Burts *et al.*, 2005). The *ess* loci of *S. aureus* MRSA252 and *S. lugdunensis* lack the genes encoding the cytoplasmic protein EsaC and the effector EsxB, but only *S. lugdunensis* N920143 contains a frameshift in the gene encoding the membrane-associated protein EssC. Among the sequenced staphylococci, only *S. epidermidis* and *S. lugdunensis* share a 12.5-kb CRISPR region, which is known to limit horizontal gene transfer (Marraffini & Sontheimer, 2008) and might give an explanation for the low number of mobile genetic elements within the *S. lugdunensis* genome.

**Table 1:** Summary of notable *Staphylococcus lugdunensis* features and distribution of orthologues in other staphylococci

	*S. lugdunensis* N920143	*S. lugdunensis* HKU09-01	*S. aureus* MRSA252	*S. epidermidis* RP62a	*S. haemolyticus*	*S. saprophyticus*	*S. carnosus*	*M. caseolyticus*
NRPS 1	+	+	+	+	−	−	−	−
NRPS 2	+	+	−	−	−	−	−	−
NRPS 3	+	+	−	−	−	−	−	−
*isd* locus	+	Duplicated	+	−	−	−	−	−
*sst* locus	Duplicated	Duplicated	+	+	+	+	+	+
*cap* locus (PS capsule)	+	+	+	−	+	+	+	+
*cap* locus (PGA capsule)	+	+	−	+	+	+	−	−
*esx* locus	+ one gene FS	+ one gene FS	+	−	−	−	−	−
Streptolysin S-like toxin	+ one gene FS	+ one gene FS	− (RF122)	−	−	−	−	−
Lantibiotic resistance locus	+	+	−	−	−	−	−	−
*agr* locus	+	+	+	+	+	+	+	−
*ica* locus	+	+	+	+	−	+	−	+
*mprF/dlt*	+	+	+	+	+	+	+	+
CRISPR region	+	+	−	+	−	−	−	−
β-Haemolysin	+	+	+	+	−	−	−	−
Putative haemolysin III	+	+	+	+	+	+	+	+

FS, frameshift; NRPS, nonribosomal peptide synthetase; PGA, polyglutamic acid; PS, polysaccharide; RF122, sequenced *Staphylococcus aureus* bovine isolate.

Because *S. lugdunensis* is more virulent than other CoNS in its ability to cause SSSTIs and IE it is worthwhile to examine the differences in the repertoires of virulence factors. The *S. aureus* sphingomyelinase β-toxin (*hlb*) is conserved in *S. lugdunensis* and a putative haemolysin III is encoded as well. However, *S. lugdunensis* does not have genes encoding coagulase, protein A, superantigens, exfoliatins, β-barrel pore-forming toxins (*hly, luk, hlg*) or small secreted proteins involved in immune evasion viz *map, efb, chp, scn, sak, ssl*.

### The *isd* locus

**Fig. 2:**
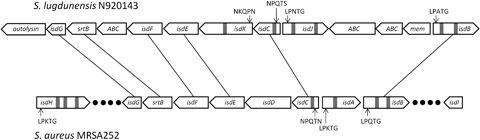
Comparison of the *isd* loci of *Staphylococcus aureus* and *Staphylococcus lugdunensis*. A schematic diagram of the *isd* loci is shown. The open boxes denote individual genes and the arrows the direction of their transcription. Encoded NEAT motifs are shown as small black boxes. Orthologous genes are linked by thin black lines. The % identities between the encoded proteins are as follows: IsdB 36.8%, IsdC 57.6%, IsdE, 74.7%, IsdF 57.7%, SrtB 58.2%, IsdG 68.2%. Cell wall sorting signals are indicated. The *isdH* and *isdI* genes of *S. aureus* are located outside the locus as indicated by black circles.

**Fig. 3:**
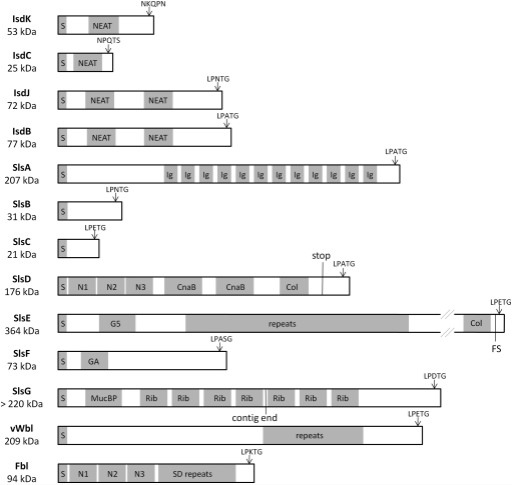
Schematic diagrams of the *Staphylococcus lugdunensis* MSCRAMMs. Predicted domains are indicated as grey boxes. NEAT, NEAT domain; GA, GA module; Ig, immunoglobulin-like fold; Col, collagen triple helix repeat; G5, G5 domain; MucBP, mucin-binding domain; Rib, Rib-like repeat; SD, serine–aspartate; FS, frame shift; see text for predicted functions of the various domains. S-labeled boxes indicate the putative signal sequences.

*Staphylococcus lugdunensis* is unique among CoNS by having a locus encoding iron-regulated surface determinant (Isd) proteins that have the potential to extract haem from haemoglobin and transport it across the cell wall using a series of wall-anchored proteins bearing near iron transporter (NEAT) motifs and into the cytoplasm using an ABC transporter. There, haem monooxygenases cleave the porphyrin ring to release the Fe^2+^. *Staphylococcus aureus* specifies three surface-exposed proteins that are anchored to peptidoglycan by processing at the C-terminal LPXTG-motif by sortase A. The IsdH, IsdB and IsdA proteins have three, two and one NEAT motifs, respectively. *Staphylococcus lugdunensis* has two putative LPXTG-anchored proteins, both with two NEAT motifs (Fig. 2 shows the *isd* loci of *S. aureus* and *S. lugdunensis* in comparison; Fig. 3 shows schematic diagrams of the surface-anchored proteins). One is an orthologue of IsdB with the sequence similarity to IsdB NEAT motifs being particularly high (50% and 55% identities). The second LPXTG-anchored protein named IsdJ has two NEAT motifs with 50% and 54% identity to the single NEAT motif of IsdA. The *S. aureus* Isd locus includes a novel sortase (SrtB) that recognizes the C-terminal NPQTN motif in IsdC when anchoring the protein to peptidoglycan. *Staphylococcus lugdunensis* has SrtB and IsdC orthologues and encodes a second putative SrtB substrate (IsdK) carrying a NKQPN motif in a gene located between *isdC* and *isdE* that replaces *isdD* in the *S. aureus* locus. In contrast to IsdD, the *S. lugdunensis* IsdK protein has a NEAT motif. Another major difference between *S. aureus* and *S. lugdunensis* Isd is the absence of the IsdI haem oxygenase and the cell wall-anchored protein IsdH. Finally, a putative autolysin is encoded downstream of *isdG* in the *S. lugdunensis* operon. No orthologue gene is present in *S. aureus*. Otherwise the overall gene organization is very similar apart from the presence of an insertion of genes encoding a membrane transporter located between the *isdA*-like gene and the *isdB* orthologue. Also a gene encoding an ABC transporter subunit is located between *isdF* and *srtB*. Whether these genes are Fur-regulated and whether they transport haem or another substrate is an open question.

Of special interest is the location of the Isd locus with respect to the replication origin of the chromosome. The *S. aureus* MRSA252 locus is located in the middle of the chromosome (nucleotide 1146876–1155059). In contrast, the *S. lugdunensis* locus (nucleotide 96442–111616) is located in a different genomic context, close to the origin of replication.

### Surface-anchored proteins

Proteins that are covalently anchored to the cell wall surface by sortase A-mediated processing of LPXTG are of particular interest because of their possible roles in adhesion to skin and host tissue, immune evasion and biofilm formation. Apart from the two Isd proteins described above, the previously described fibrinogen-binding protein Fbl that is related to ClfA of *S. aureus* (Geoghegan *et al.*, 2010) and the von Willebrand factor-binding protein vWbl (Nilsson *et al.*, 2004), *S. lugdunensis* has seven genes with the potential to encode wall-anchored proteins ranging in size from 20.8 to 380 kDa. These proteins have been called *S. lugdunensis* surface proteins (Sls). Figure 3 shows schematic diagrams of these proteins. Analysis using the Pfam database (Sammut *et al.*, 2008) has revealed several interesting features. (1) The 1930 residue SlsA protein has 12 nonidentical repeats of an IgG-like fold located between the LPXTG sequence and the 734 residue N-terminal domain. (2) SlsE (3459 residues) has an 1240-residue N-terminal domain containing a 78-residue G5 motif (Ruggiero *et al.*, 2009) followed by a collagen-like sequence (24 residues), 46 repeats of a 31 residue motif and a C-terminal collagen-like sequence (50 residues). The *slsE* gene of N920143 has a frameshift between the region encoding the collagen-like domain and the LPXTG sequence while that of HKU09-01 is intact. (3) SlsG has a 874-residue N-terminal domain with a 125-residue MucBP domain (Du *et al.*, 2011) followed by repeats of a Rib-like domain (Wastfelt *et al.*, 1996) seen in group B streptococci surface-anchored proteins. There are 18 Rib repeats in the HKU09-01 protein, whereas in the N920143 protein the number of repeats is uncertain because the gene is located on two contigs in our sequence. (4) The SlsD protein has a 575-residue N-terminal domain that has sequence and putative structural similarity to the fibrinogen-binding domain of SdrG of *S. epidermidis* (Ponnuraj *et al.*, 2003). Located C-terminally to this are two repeats with similarity to the B repeats of the collagen-binding protein CNA of *S. aureus* and a 45-residue collagen-like domain. The *slsD* gene contains a nonsense codon located just 5′ to the region encoding LPXTG so it is unlikely that the protein would remain anchored to the cell wall. (5) The 659-residue SlsF protein has a 41-residue GA albumin-binding domain similar to that of *Peptostreptococcus magnus* (Johansson *et al.*, 1997).

All MSCRAMMs are highly conserved in the strain HKU09-01 with only minor differences in the number of repeats within the stalk regions. The only major difference is that *slsE* does not contain a frameshift in HKU09-01. Although both strains represent isolates form distant geographical origins, the nonsense mutation in *slsD* is present in both strains.

## Discussion

The first in-depth analysis of an *S. lugdunensis* genome sequence and the comparison with several other staphylococci revealed a multitude of interesting characteristics, making *S. lugdunensis* an outstanding member of the staphylococci. The core genome of all species included in the evaluation is highly conserved and encodes housekeeping functions like DNA replication, RNA synthesis, sugar and amino acid degradation/biosynthesis and metabolite transport. However, several features are apparent in *S. lugdunensis* that make it unique and place it in between *S. aureus* and the other CoNS. The presence of three nonribosomal peptide synthetases is remarkable, considering that most staphylococci encode not a single one. One can only speculate about the products, but they might support *S. lugdunensis* while colonizing the human skin by inhibiting other skin commensals or by facilitating the uptake of rare ions or other substrates. Furthermore *S. lugdunensis* encodes a plethora of surface-anchored proteins with sizes and domain organizations unusual for staphylococci.

*Staphylococcus lugdunensis* is known to be an important pathogen although it causes invasive infections only infrequently. Paradoxically, once *S. lugdunensis* gets established in a thrombus on a heart valve or is encased in an abscess it appears to be as virulent as *S. aureus* (Frank *et al.*, 2008). The genome sequence revealed only very few putative virulence factors, verifying earlier reports about the absence of many typical *S. aureus* toxin genes in *S. lugdunensis* (Fleurette *et al.*, 1989). This might explain the low infectivity of the organism. However, *S. lugdunensis* has the potential to encode a streptolysin S-like toxin and secretion system, which is well described for different bacterial species, but among the staphylococci it is found only in the bovine *S. aureus* isolate RF122 (Lee *et al.*, 2008). Further, *S. lugdunensis* is the only CNS carrying an ESAT-6 system similar to the one encoded by *S. aureus*. A striking difference from *S. aureus* is the absence of any obvious immune evasion molecules. In *S. aureus*, immune evasion molecules are located on mobile genetic elements, pathogenicity islands or prophages. The lack of these elements seems to be a characteristic for *S. lugdunensis* and might in part explain its low infectivity.

The most interesting linkage between *S. aureus* and *S. lugdunensis* is the presence of an *isd* locus. In *S. aureus*, the locus has been shown to be important for pathogenicity. Isd proteins of *S. aureus* are not just involved in the acquisition of iron. In particular, IsdA is an important virulence factor because its C-terminal part confers resistance to antimicrobial fatty acids and lantibiotics (Clarke *et al.*, 2007). Furthermore IsdA binds to fibronectin, fibrinogen, lactoferrin, transferrin, feutin, involucrin, loricrin and cytokeratin 10 and is important for nasal colonization (Clarke *et al.*, 2004, 2009; Clarke & Foster, 2008). IsdB promotes binding to and activation of platelets (Miajlovic *et al.*, 2010). Interestingly, IsdB and the N-terminal domain of IsdA are conserved in *S. lugdunensis*, perhaps suggesting similar functions of the proteins. In addition, the duplication of the locus in HKU09-01 strengthens the hypothesis of an important function of the locus. The ability of the bacterium to adhere to certain ligands or to resist against antimicrobial agents might be enhanced by a gene dosage effect.

In *S. lugdunensis* and *S. aureus*, the Isd loci are located in different chromosomal contexts, suggesting that they were acquired independently from different sources. This explains the apparent differences between the loci including the absence of *isdI* and *isdG* in *S. lugdunensis*. In *S. aureus* these genes are located outside the *isd* operon and must have been acquired independently of the other genes to support the function of the operon. The functions of these proteins are probably dispensable in *S. lugdunensis*. However, the presence of an independently acquired *isd* operon in *S. lugdunensis* and *S. aureus* suggests convergent evolution towards invasive behaviour that has not been described for any other CoNS.

The conclusions of the genome analysis give a picture showing *S. lugdunensis* to be a skin commensal that is well equipped for the survival and competition on human skin. Nevertheless, provided with capsules, different toxins, putative haemolysins and a plethora of surface-anchored proteins, the aggressive, *S. aureus-*like behaviour of *S. lugdunensis* that distinguishes it from the other CoNS might be explained. The properly annotated sequence provides the starting point for projects to investigate the mechanistic basis of skin colonization and pathogenesis by facilitating cloning and expression of genes for biochemical studies, and for generating site-specific mutants by allelic exchange to allow testing of Koch's Postulates at the molecular level.

## Statement

Data deposition: The sequence and annotation of the N920143 genome has been deposited in the EMBL database under the accession number FR870271.
